# Association of low‐activity ALDH2 and alcohol consumption with risk of esophageal cancer in Chinese adults: A population‐based cohort study

**DOI:** 10.1002/ijc.31566

**Published:** 2018-06-11

**Authors:** Canqing Yu, Yu Guo, Zheng Bian, Ling Yang, Iona Y. Millwood, Robin G. Walters, Yiping Chen, Yan Chen, Xi Zhang, Yulong Lei, Junshi Chen, Zhengming Chen, Jun Lv, Liming Li, on behalf of the China Kadoorie Biobank Collaborative Group

**Affiliations:** ^1^ Department of Epidemiology and Biostatistics, School of Public Health Peking University Health Science Center Beijing China; ^2^ Chinese Academy of Medical Sciences Beijing China; ^3^ Clinical Trial Service Unit & Epidemiological Studies Unit (CTSU), Nuffield Department of Population Health University of Oxford Oxford United Kingdom; ^4^ Hainan Center for Disease Control & Prevention Haikou Hainan China; ^5^ Maiji Center for Disease Control & Prevention Tianshui Gansu China; ^6^ China National Center for Food Safety Risk Assessment Beijing China; ^7^ Peking University Institute of Environmental Medicine Beijing China

**Keywords:** alcohol drinking, acetaldehyde dehydrogenase 2, alcohol flushing response, esophageal neoplasms, prospective studies

## Abstract

Existing evidence remains inconclusive as to how the association between inactive ALDH2 and esophageal cancer (EC) depends on alcohol consumption. The study is based on the China Kadoorie Biobank cohort, with 10 years follow‐up of 0.5 million adults aged 30–79 years. ALDH2 activity was assessed by both self‐reported flushing response and Glu504Lys (rs671 G > A) polymorphism. Among both male and female participants who consumed alcohol less than weekly (*n* = 69,519; 211 EC cases), low active or inactive ALDH2 was not associated with increased EC risk [HRs (95% CIs): GA *vs*. GG 0.75 (0.54, 1.04); AA *vs*. GG 1.01 (0.46, 2.20)]. Among male weekly alcohol consumers, both flushing response [*n* = 59,380; 501 EC cases; HRs (95% CIs): “soon after drinking” *vs*. “no” flushing response 1.45 (1.05, 2.01)] and rs671 [*n* = 10,692; 94 EC cases; GA *vs*. GG 3.31 (1.94, 5.67)] were associated with EC risk. The increased EC risk associated with “soon” response or rs671 GA was apparent in men consuming alcohol ≥30g/d. Among male daily consumers, the HRs (95% CIs) for EC associated with 15g/d of alcohol were 1.28 (1.15, 1.44) for “soon” response [*vs*. other responses: 1.12 (1.09, 1.15); *p*_interaction_ = 0.047; *n* = 36,401, 425 EC cases] and 1.41 (1.08, 1.82) for rs671 GA [*vs*. GG: 1.16 (1.06, 1.27); *p*_interaction_ = 0.493; *n* = 6,607, 80 EC cases]. Self‐reported flushing response had low sensitivity (56.8%) and high specificity (88.4%) in identifying rs671 A allele among male weekly alcohol consumers. In conclusion, low‐activity ALDH2 was associated with increased EC risk among male heavy alcohol consumers. More accurate measurement of alcohol‐related EC risk allows better achievement of precision prevention.

## Abstract 2

**What's new?**

Acetaldehyde is a toxic metabolite considered a major cause of alcohol‐induced carcinogenesis. The key enzyme for acetaldehyde elimination is acetaldehyde dehydrogenase 2 (ALDH2), and an association between *ALDH2* genotype and EC risk that is dependent on alcohol consumption has been suggested. The evidence remains inconclusive, however. In this large prospective cohort of Chinese adults, the *ALDH2* rs671 A allele was not associated with increased EC risk in the absence of alcohol consumption. The increased EC risk associated with low‐activity ALDH2, characterized as self‐reported flushing response or rs671 GA, was apparent in men consuming alcohol ≥30g/day, but not among light‐to‐moderate consumers.

**Abbreviations**
ALDH2: acetaldehyde dehydrogenase 2
CI: confidence interval
CKB: China Kadoorie Biobank
EC: esophageal cancer
ESCC: esophageal squamous cell cancer
HRs: hazard ratios
IARC: International Agency for Research on Cancer
ICD: International Classification of Diseases

## Introduction

Esophageal cancer (EC) remains a global concern because of its increasing incidence and persistently poor survival.^1^, ^2^ China is among the highest EC incidence countries.^2^ Alcohol consumption is a well‐established risk factor for esophageal squamous cell cancer (ESCC),^3^ the most common histological subtype globally.^2^ Acetaldehyde, a toxic metabolite of alcohol that damages DNA, has been classified as a Group 1 human carcinogen by the International Agency for Research on Cancer (IARC) and is considered a major cause underlying alcohol‐induced carcinogenesis.^4^

The key enzyme for acetaldehyde elimination is acetaldehyde dehydrogenase 2 (ALDH2), encoded by the *ALDH2* gene.^5^ A Glu504Lys polymorphism in *ALDH2* which reduces its activity is almost exclusively present and highly prevalent among East Asians.^6^ In carriers of ALDH2 Lys/Lys and Glu/Lys, the enzyme activity is nearly 0% and 17–38% of the normal activity, respectively.^7^ This dramatic reduction in enzyme activity leads to accumulation of acetaldehyde in the circulation even after moderate alcohol consumption.^8^ The ALDH2 Lys variant also causes the well‐known Asian flush, an unpleasant physiological response to alcohol consumption that includes facial flushing, nausea and tachycardia and inhibits alcohol consumption.^9^

It has been suggested that there is an association between *ALDH2* genotype and EC risk which is dependent on alcohol consumption. In a meta‐analysis of 31 case–control studies, the possession of inactive ALDH2 does not increase EC risk unless alcohol is consumed.^10^ Numerous case–control studies, almost exclusively conducted in Asian populations, have addressed the association of EC with *ALDH2* genotype or self‐reported facial flushing as a surrogate marker of inactive ALDH2.^10^, ^11^ These studies presented substantial heterogeneity in the definition of alcohol consumption categories and the control of confounding, and were particularly vulnerable to recall bias and reverse causality due to change of consumption habits following their symptoms of prediagnostic EC. Two prospective studies conducted in Japanese men, with 33 and 215 incident EC cases separately, yielded inconsistent results.^12^, ^13^ Thus, the existing evidence remains inconclusive as to the association between inactive ALDH2 and EC risk. More prospective studies are warranted to examine the joint effects of ALDH2 deficiency and amount of alcohol consumed on EC and quantify the dose–response relationship between alcohol consumption and EC by *ALDH2* genotype.

In the China Kadoorie Biobank (CKB) prospective study, we first examined whether *ALDH2* genotype was associated with EC risk in the absence of alcohol consumption in both men and women. We further examined the effects of self‐reported flushing response, *ALDH2* genotype and their joint effects with alcohol consumption on EC risk in male participants. We did not include women in this analysis because of their very low prevalence of alcohol consumption. We also assessed the sensitivity and specificity of self‐reported flushing response for identifying inactive ALDH2 in this Chinese population.

## Materials and Methods

### Study population

The CKB is a cohort established in 10 study areas geographically spread across China, including five urban and five rural areas. During 2004–2008, we enrolled 512,891 adults aged 30–79 years. Trained staff collected socio‐demographic characteristics, lifestyle behaviors and medical history using a laptop‐based questionnaire and took physical measurements using calibrated instruments. Further detailed description is available elsewhere.^14^, ^15^ All participants provided written informed consent. The Ethical Review Committee of the Chinese Center for Disease Control and Prevention (Beijing, China) and the Oxford Tropical Research Ethics Committee, University of Oxford (UK) approved the study.

### Assessment of alcohol consumption and flushing response

In the baseline questionnaire, participants reported their usual frequency of alcohol consumption (never, only occasionally, only at certain seasons, monthly but less than weekly or at least once a week) during the past 12 months. Participants who consumed alcohol at least once a week were asked how many days they drank in a typical week (1–2, 3–5 or 6–7 days), the type of alcoholic beverage consumed habitually (beer, rice wine, wine or Chinese spirit with low or high alcohol content), the amount of alcohol consumed on a typical drinking day, and the age they started consuming alcohol weekly. Based on this information, ethanol in grams consumed on a typical drinking day was calculated.^16^ Weekly consumers were also asked: “After consuming alcohol, do you usually experience facial flushing, tachycardia or dizziness?” (question A), with options of (1) yes, soon after first mouthful (hereinafter abbreviated to “soon”), (2) yes, after consuming a small amount of alcohol (“small”), (3) yes, but only after consuming a large amount of alcohol (“large”) or (4) no. Among 1,952 weekly alcohol consumers who completed the same questionnaire twice at a median interval of 2.6 years between baseline and the first re‐survey,^14^ spearman's correlation coefficient was 0.56 for alcohol consumption frequency, 0.58 for the amount of alcohol consumed, 0.25 for flushing response, indicating a moderate reliability of the measures of alcohol consumption patterns.

In the second re‐survey during 2013–2014, participants were asked about their past flushing status given that some individuals can become tolerant to the flushing effect.^9^ Participants who answered “no” or “large” for current flushing response (question A) were asked: “Did you experience facial flushing, tachycardia, or dizziness in the first one or two years after you started consuming alcohol regularly?” (question B). Participants who answered “soon” or “small” to question A were considered to be “current flushing;” those who answered “soon” or “small” to question B but not to question A were considered to be “former flushing.”

### Assessment of *ALDH2* genotype

Genotyping using a 384‐SNP Illumina® GoldenGate array, including *ALDH2* rs671 G > A (Glu504Lys), was done in 95,680 randomly selected CKB participants at the BGI laboratory in Shenzhen, China. A total of 93,141 participants passed quality control (call rate ≥98%, no sex mismatch, heterozygosity F statistic SD score <5). SNPs with low call rate (<95%) or Hardy–Weinberg disequilibrium (*p* < 0.05/384 = 1.3 × 10^−4^) were excluded. Mean genotyping concordance was 99.98% (range: 98.66–100%) based on 2,063 duplicate samples included for quality control purposes. The call rate of rs671 was ≥99.9%, that is, 93,129 participants had genotype information.

### Ascertainment of EC cases

We ascertained incident EC cases since the participants' enrollment into the study at baseline by linking to local disease and death registries, to the national health insurance system, and by active follow‐up.^14^ Trained staff, blinded to the baseline information, coded all cases using the 10th revision of the International Classification of Diseases (ICD‐10). For the present analysis, EC cases were defined by ICD‐10 code C15.

Retrieving medical records of incident EC cases is in process in the CKB study. Trained staff reviews medical records for diagnosis validation and further collection of detailed clinical information such as pathology subtype. Up to now, we have retrieved medical records for 870 newly reported EC cases during follow‐up, among which 843 (96.9%) were confirmed as EC cases, and 65.4% (569/843 cases) had pathology reports. After excluding 37 cases with subtype reported as “unknown,” 91.9% (489/532 cases) were ESCC.

### Statistical analysis

**Figure 1:**
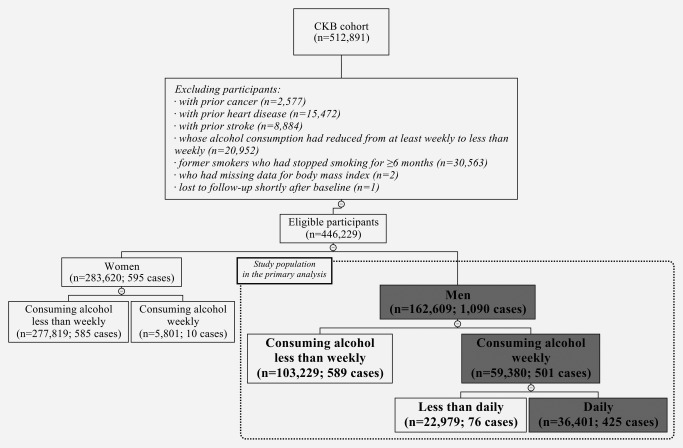
Flowchart of participants included in the analysis of self‐reported flushing response and esophageal cancer risk. The number of eligible participants and incident esophageal cancer cases is indicated in parentheses. The boxes with gray background indicate the subsets of participants used in the primary analysis.

As presented in the flowchart of participants (Fig. 1), 446,229 participants were eligible for analysis of the self‐reported flushing response after excluding those with prior cancer, and those with cardiovascular diseases or who had quit consumption of alcohol or tobacco at baseline to avoid misleadingly elevated risk for the reference group. Only 5,801 (2.0%) women consumed alcohol weekly. We, therefore, only included 162,609 men in the present analysis.

**Figure 2:**
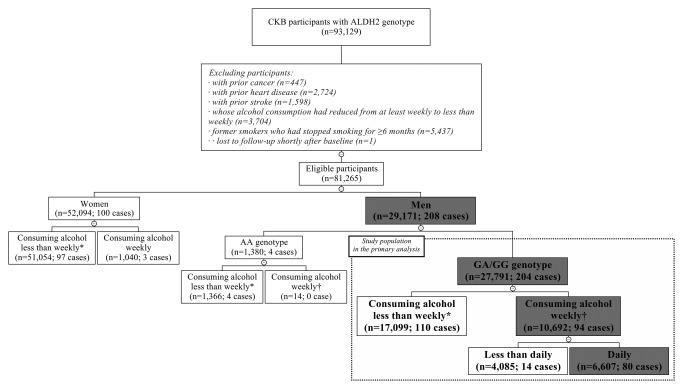
Flowchart of participants included in the analysis of *ALDH2* genotype and esophageal cancer risk. The number of eligible participants and incident esophageal cancer cases is indicated in parentheses. The boxes with gray background indicate the subsets of participants used in the primary analysis. *A total of 69,519 male and female participants who consumed alcohol less than weekly, with 211 incident esophageal cancer cases, were used to analyze the association between *ALDH2* genotype and esophageal cancer risk. ^†^A total of 10,706 male weekly alcohol consumers, with 94 incident esophageal cancer cases, were used to analyze the sensitivity and specificity of self‐reported flushing response.

For *ALDH2* genotype analysis, 81,265 eligible participants had genotyping data (Fig. 2). We analyzed the association of *ALDH2* genotype with EC risk among 69,519 male and female participants who consumed alcohol less than weekly, excluding the influence of alcohol consumption. The analysis of *ALDH2* genotype and alcohol consumption on EC risk was confined to 27,791 men with rs671 GA/GG because only 14 (1.0%) men with AA consumed alcohol weekly. We present the results for the full dataset, but the exclusion of 4,110 participants due to first‐degree relationships had no appreciable effect on the results.

Due to a small number of EC cases among both less‐than‐daily and <30 g/d of alcohol consumers, yielding results with very wide confidence interval (CI) (data not shown), we combined these two groups of alcohol consumers into one category in the present analysis. We calculated person‐years at risk from the baseline date to the diagnosis of EC, death, loss to follow‐up, or December 31, 2015, whichever came first. The loss to follow‐up in the CKB study refers to a participant whose permanent registered residence has moved out of the jurisdiction of the Regional Coordinating Center. By December 31, 2015, of all 512,891 participants, 37,289 (7.3%) died and 4,875 (<1%) were lost to follow‐up. We used Cox proportional hazards model to estimate the hazard ratios (HRs) and 95% CIs, with age as the underlying time scale and stratified jointly by study area and age at baseline in the 5‐year interval. Test and graph based on Schoenfeld residuals showed that the proportional hazards assumption was satisfied.

The multivariable model was adjusted for age, education, marital status, household income, tobacco smoking, hot tea consumption, physical activity, intakes of red meat, fresh fruits and vegetables, and preserved vegetables, body mass index, waist‐to‐hip ratio, family history of cancer, and the frequency and amount of alcohol consumption. We examined whether the association of EC risk with self‐reported flushing response or *ALDH2* genotype differed by the amount of alcohol consumed per day, and whether the association of EC risk with alcohol consumption differed by the flushing response or *ALDH2* genotype, by using likelihood ratio test comparing models with and without cross‐product term. We also estimated the population attributable fraction (PAF) as P×[(HR_adj_‐1)/HR_adj_], where P is the proportion of EC cases having exposure of interests, and HR_adj_ is the multivariable‐adjusted relative risk for this exposure category relative to the unexposed group.^17^

We also assessed the questions for detecting inactive ALDH2 in terms of sensitivity (the proportion of participants possessing rs671 A allele who were correctly identified as a flusher by the questionnaire) and specificity (the proportion of participants possessing active rs671 GG genotype who were correctly identified as a non‐flusher by the questionnaire) among male weekly alcohol consumers.

We performed all statistical analyses using Stata (version 14.2, StataCorp, College Station, Texas USA).

### Role of the funding source

The funders had no role in the study design, data collection, data analysis and interpretation, writing of the report or the decision to submit the article for publication.

## Results

### Baseline characteristics of eligible male participants

Of 162,609 men aged 50.9 ± 10.6 years, 36.5% of them consumed alcohol weekly (Supporting Information Table S1). Male daily consumers who consumed alcohol ≥30 g/d were more likely to be rural residents, smoke and consume hot tea. Heavy alcohol consumers who reported “no” or “large” for flushing response usually consumed more alcohol per day than those who reported “soon” or “small.” Of 29,171 men who were genotyped for rs671, those with AA or GA genotypes accounted for 37.7%. Compared to men with rs671 GG, those with A alleles were less likely to be daily alcohol consumers and consumed less alcohol on a typical drinking day.

### Sensitivity and specificity of self‐reported flushing response

When only current flushing (question A) was used to detect inactive ALDH2, the sensitivity and specificity for identification were 56.8% and 88.4% among 10,706 male weekly alcohol consumers (Supporting Information Table S2). Adding former flushing response (question B) to the screening did not appreciably improve the results.

### Self‐reported flushing response and EC

During a median 9.2 years (4.0 million person‐years) of follow‐up among 446,229 participants, there were 1,090 incident EC cases in men and 595 in women. Self‐reported flushing “soon” after drinking was associated with increased risk of EC among 59,380 male weekly alcohol consumers (501 incident EC cases) (Table 1). After adjustment for the amount of alcohol consumed and other potential confounders, the adjusted HRs (95% CIs) for EC were 0.96 (0.79, 1.18) for those reporting “large,” 1.19 (0.88, 1.61) for “small” and 1.45 (1.05, 2.01) for “soon,” compared to men reporting “no” flush response.

**Table 1:** HRs (95% CIs) for esophageal cancer according to self‐reported flushing response or *ALDH2* genotype among male weekly alcohol consumers

	Cases	PYs	Cases/PYs (/1,000)	Age‐adjusted	Multivariable‐adjusted^a^	Further adjusted for alcohol consumption^b^
Self‐reported flushing response (*n* = 59,380)						
No	205	227,450	0.90	1.00	1.00	1.00
Large	191	211,232	0.90	0.95 (0.78, 1.17)	0.99 (0.81, 1.21)	0.96 (0.79, 1.18)
Small	57	52,688	1.08	0.98 (0.73, 1.33)	1.02 (0.75, 1.38)	1.19 (0.88, 1.61)
Soon	48	43,379	1.11	1.10 (0.80, 1.51)	1.15 (0.84, 1.59)	1.45 (1.05, 2.01)
*ALDH2* genotype (*n* = 10,692)						
GG	73	81,027	0.90	1.00	1.00	1.00
GA	21	15,125	1.39	2.35 (1.42, 3.89)	2.61 (1.55, 4.39)	3.31 (1.94, 5.67)

Abbreviations: HR: hazard ratio; CI: confidence interval; PYs: person‐years; MET: metabolic equivalent of task.

a Multivariable model was adjusted for age (year), education (no formal school, primary school, middle school, high school, college or university or above), marital status (married, widowed, divorced/separated or never married), household income (RMB/year: <2,500, 2,500–4,999, 5,000–9,999, 10,000–19,999, 20,000–34,999 or ≥35,000), tobacco smoking (nonsmokers, current smokers 1–9, 10–19, 20–29 or ≥30 cigarettes or equivalents per day), tea consumption and temperature preference (consuming tea less than weekly, weekly or daily; further categorized into preferring warm, hot or burning hot tea among daily consumers), physical activity (MET‐hr/day), intakes of red meat, fresh fruits and vegetables and preserved vegetables (day/week, calculated by assigning participants to the midpoint of their intake category), body mass index (kg/m^2^), waist‐to‐hip ratio and family history of cancer (presence or absence).

b Multivariable model was further adjusted for frequency of alcohol consumption (1–2 days/week, 3–5 days/week or 6–7 days/week) and the amount of alcohol consumed on a typical drinking day (g).

### *ALDH2* genotype and EC

Among 10,692 male weekly alcohol consumers (94 EC cases), rs671 GA was associated with increased risk of EC, with an adjusted HR (95% CI) of 3.31 (1.94, 5.67) compared to GG (Table 1). There was no association between *ALDH2* genotype and EC risk among 69,519 male and female participants who consumed alcohol less than weekly, 211 of whom developed EC during follow‐up. Compared to participants with rs671 GG, the multivariable‐adjusted HRs (95% CIs) for EC were 0.75 (0.54, 1.04) and 1.01 (0.46, 2.20) for those with GA and AA, respectively.

### Flushing response, *ALDH2* genotype with alcohol consumption on EC

Among male weekly alcohol consumers, when stratified by the amount of alcohol consumed, the statistically significant increase in EC risk associated with “soon” flushing response was present in daily consumers of ≥60 g/d of alcohol, and increased EC risk associated with rs671 GA present in those of ≥30 g/d of alcohol. However, there was no statistically significant difference in the association of EC risk with self‐reported flushing response (*p*_interaction_ = 0.618) or *ALDH2* genotype (*p*_interaction_ = 0.376) across the amount of alcohol consumed per day (Table 2). EC risk increased with alcohol consumption for all male daily alcohol consumers (Table 3). However, heavier alcohol consumption was associated with greater increase in EC risk for those with “soon” flushing response or rs671 GA. The HRs (95% CIs) for EC risk associated with 15 g of alcohol per day were 1.28 (1.15, 1.44) for men with “soon” response compared to 1.12 (1.09, 1.15) for those with other response (*p*_interaction_ = 0.047), and 1.41 (1.08, 1.82) for men with rs671 GA compared to 1.16 (1.06, 1.27) for those with GG (*p*_interaction_ = 0.493).

**Table 2:** HRs (95% CIs) for esophageal cancer in relation to self‐reported flushing response or *ALDH2* genotype stratified by the amount of alcohol consumed per day among male weekly alcohol consumers

	Less than daily or <30 g/d	30–59 g/d	60–89 g/d	≥90 g/d
Self‐reported flushing response (*n* = 59,380); *p*_interaction_ = 0.618				
No. of participants	30,624	11,914	10,287	6,555
No				
Cases	54	42	48	61
Cases/PYs (/1,000)	0.47	0.86	1.23	2.41
HRs (95% CIs)^a^	1.00	1.00	1.00	1.00
Large				
Cases	36	36	49	70
Cases/PYs (/1,000)	0.34	0.92	1.27	2.49
HRs (95% CIs)^a^	0.72 (0.46, 1.11)	1.06 (0.66, 1.69)	1.06 (0.70, 1.62)	1.07 (0.75, 1.53)
Small				
Cases	16	15	13	13
Cases/PYs (/1,000)	0.54	1.43	1.39	4.19
HRs (95% CIs)^a^	1.14 (0.64, 2.03)	2.10 (1.12, 3.92)	0.99 (0.52, 1.87)	1.22 (0.66, 2.28)
Soon				
Cases	13	11	14	10
Cases/PYs (/1,000)	0.46	1.37	2.56	6.15
HRs (95% CIs)^a^	1.01 (0.54, 1.90)	1.62 (0.81, 3.24)	1.91 (1.03, 3.56)	2.45 (1.23, 4.91)
*ALDH2* genotype (*n* = 10,692); *p*_interaction_ = 0.376				
No. of participants	5,436	2,161	1,888	1,207
GG				
Cases	17	16	18	22
Cases/PYs (/1,000)	0.43	0.97	1.18	2.21
HRs (95% CIs)^a^	1.00	1.00	1.00	1.00
GA				
Cases	5	4	7	5
Cases/PYs (/1,000)	0.50	1.43	4.29	6.45
HRs (95% CIs)^a^	2.38 (0.64, 8.84)	6.42 (1.01, 40.79)	6.33 (1.85, 21.68)	3.38 (1.04, 10.92)

Abbreviations: HR: hazard ratio; CI: confidence interval; PYs: person‐years; MET: metabolic equivalent of task.

a Multivariable model was adjusted for age (year), education (no formal school, primary school, middle school, high school, college or university or above), marital status (married, widowed, divorced/separated or never married), household income (RMB/year: <2,500, 2,500–4,999, 5,000–9,999, 10,000–19,999, 20,000–34,999 or ≥35,000), tobacco smoking (nonsmokers, current smokers 1–9, 10–19, 20–29 or ≥30 cigarettes or equivalents per day), tea consumption and temperature preference (consuming tea less than weekly, weekly or daily; further categorized into preferring warm, hot or burning hot tea among daily consumers), physical activity (MET‐hr/day), intakes of red meat, fresh fruits and vegetables and preserved vegetables (day/week, calculated by assigning participants to the midpoint of their intake category), body mass index (kg/m^2^), waist‐to‐hip ratio and family history of cancer (presence or absence).

**Table 3:** HRs (95% CIs) for esophageal cancer in relation to the amount of alcohol consumed per day stratified by the self‐reported flushing response or *ALDH2* genotype among male daily alcohol consumers

	<30 g/d	30–59 g/d	60–89 g/d	≥90 g/d	per 15 g/d
Self‐reported flushing response (*n* = 36,401); *p*_interaction_ = 0.047					
Small/large/no					
Cases	40	93	110	144	–
Cases/PYs (/1,000)	0.66	0.94	1.27	2.55	–
HRs (95% CIs)^a^	1.00	1.35 (0.92, 1.98)	1.83 (1.23, 2.71)	3.67 (2.48, 5.44)	1.12 (1.09, 1.15)
Soon					
Cases	3	11	14	10	–
Cases/PYs (/1,000)	0.39	1.37	2.56	6.15	–
HRs (95% CIs)^a^	1.00	8.13 (1.28, 51.44)	27.05 (3.82, 191.71)	48.81 (6.42, 370.93)	1.28 (1.15, 1.44)
*ALDH2* genotype (*n* = 6,607); *p*_interaction_ = 0.493					
GG					
Cases	4	16	18	22	–
Cases/PYs (/1,000)	0.44	0.97	1.18	2.21	–
HRs (95% CIs)^a^	1.00	1.80 (0.53, 6.10)	2.32 (0.66, 8.23)	4.87 (1.37, 17.38)	1.16 (1.06, 1.27)
GA					
Cases	4	4	7	5	–
Cases/PYs (/1,000)	1.43	1.43	4.29	6.45	–
HRs (95% CIs)^a^	1.00	0.74 (0.07, 8.00)	12.86 (0.64, 259.44)	51.04 (1.44, 1,807.71)	1.41 (1.08, 1.82)

Abbreviations: HR: hazard ratio; CI: confidence interval; PYs: person‐years; MET: metabolic equivalent of task.

a Multivariable model was adjusted for age (year), education (no formal school, primary school, middle school, high school, college or university or above), marital status (married, widowed, divorced/separated or never married), household income (RMB/year: <2,500, 2,500–4,999, 5,000–9,999, 10,000–19,999, 20,000–34,999 or ≥35,000), tobacco smoking (nonsmokers, current smokers 1–9, 10–19, 20–29 or ≥30 cigarettes or equivalents per day), tea consumption and temperature preference (consuming tea less than weekly, weekly or daily; further categorized into preferring warm, hot or burning hot tea among daily consumers), physical activity (MET‐hr/day), intakes of red meat, fresh fruits and vegetables and preserved vegetables (day/week, calculated by assigning participants to the midpoint of their intake category), body mass index (kg/m^2^), waist‐to‐hip ratio and family history of cancer (presence or absence).

**Figure 3:**
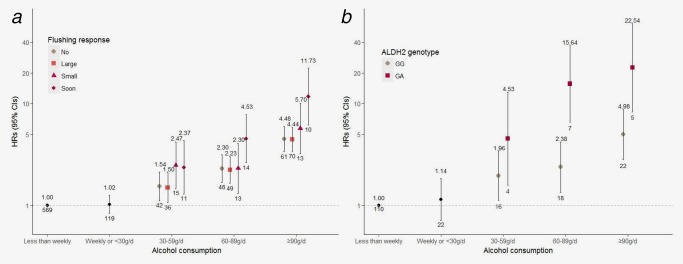
HRs (95% CIs) for joint effects of alcohol consumption with self‐reported flushing response or *ALDH2* genotype on esophageal cancer risk among male participants. HR indicates hazard ratio; and CI, confidence interval. (*a*) Self‐reported flushing response (*n* = 162,609); (*b*) *ALDH2* genotype (*n* = 27,791). The vertical lines represent 95% CI. The values above the vertical lines are the point estimates of HRs, and the values below are the numbers of EC cases. The reference group was men who consumed alcohol less than weekly. The multivariable model was adjusted for age, education, marital status, household income, tobacco smoking, tea consumption and temperature preference, physical activity, intakes of red meat, fresh fruits and vegetables and preserved vegetables, body mass index, waist‐to‐hip ratio and family history of cancer. [Color figure can be viewed at http://wileyonlinelibrary.com]

We further examined the joint effects of alcohol consumption with flushing response or *ALDH2* genotype on EC risk among all eligible male participants. Compared to men consuming alcohol less than weekly, consuming alcohol ≥30 g/d was associated with an elevated EC risk for all men with any flushing response or genotype (Fig. 3; Supporting Information Tables S3 and S4). Among men who consumed alcohol ≥90 g/d, the highest EC risk was for those with “soon” flushing response (HR = 11.73; 95% CI: 6.17, 22.31) and for those with rs671 GA (HR = 22.54; 95% CI: 8.30, 61.21).

If the exposure of interest is causal, the fraction of EC risk in the male population that would be eliminated if participants who have low‐activity ALDH2 (rs671 GA) and consume alcohol ≥30 g/d changed to be light‐to‐moderate consumers or abstain from alcohol (i.e., the population attributable fraction, PAF) was 7%.

## Discussion

In this large prospective Chinese cohort, the rs671 A allele was not associated with increased EC risk in the absence of alcohol consumption. Low‐activity ALDH2, characterized as self‐reported flushing response or rs671 GA heterozygotes, was associated with an increased EC risk among male heavy alcohol consumers of ≥30 g/d. The increased EC risk associated with 15 g of alcohol per day was 30–40% among daily alcohol consumers with low‐activity ALDH2, greater than those with active ALDH2. In addition, self‐reported flushing response had low sensitivity when used as a screening tool for inactive ALDH2 among regular alcohol consumers.

The rs671 GA heterozygotes were linked to an increased EC risk (OR = 2.34; 95% CI: 1.75, 3.13) in a recent meta‐analysis of 31 case–control studies, almost exclusively conducted in China and Japan.^10^ The OR (95% CI) of GA heterozygotes for EC was 1.21 (0.95, 1.73) among non‐alcohol consumers, 3.79 (3.04, 4.72) among light consumers (1–350 g/week of alcohol) and 6.50 (5.34, 7.92) among heavy consumers (≥350 g/week of alcohol). However, there was substantial unexplained heterogeneity in this meta‐analysis even after performing subgroup analyses. In another meta‐analysis of five case–control studies and two cohort studies, alcohol flushing response was also associated with ESCC (OR = 1.97; 95% CI: 1.25, 3.13).^11^ However, the corresponding pooled risk estimate for the subgroup analysis of cohort studies was not statistically significant (OR = 1.59; 95% CI: 0.81, 3.10).^12^, ^13^ The ORs (95% CIs) for flushers *vs*. non‐flushers were 1.02 (0.53, 1.99) among non‐ or light consumers (<200 g/week), 2.54 (1.64, 3.91) among moderate consumers (200–390 g/week) and 2.90 (1.82, 4.62) among heavy consumers (>390 g/week).^11^ Several previous Chinese studies also showed increased EC risk associated with GA heterozygotes among non‐consumers or consumers of ≤30 g/d.^10^, ^18^

In our study, we consistently observed that the effect of GA heterozygotes, genotyped directly or using facial flushing as a surrogate marker, on the EC risk was only presented among male alcohol consumers of ≥30 g/d. This result is biologically plausible. Possession of the deficient allele does not increase EC risk unless alcohol is consumed. The heterozygous carriers, due to residual ALDH2 enzyme activity, may eliminate acetaldehyde and experience a less severe response when consuming alcohol lightly; but are unable to promptly transform acetaldehyde when consuming heavily and bear the increased EC risk. The association of GA heterozygotes with EC risk among non‐consumers or light consumers seen in previous studies^10^, ^18^ is more likely to be explained by the potential bias in the measurement of alcohol consumption inherent in the case–control design.

The EC risk associated with the synergistic interaction between inactive ALDH2 and alcohol consumption underscores the importance of screening for the ALDH2 deficiency. In our study, we used a single question asking about current alcohol flushing, with low sensitivity but high specificity in identifying inactive ALDH2. The addition of past flushing response did not improve the sensitivity. One of the main reasons for the low sensitivity is that we applied the screening question only to the regular alcohol consumers, in which the vast majority had rs671 GG and GA genotypes. Alcohol flushing among GA heterozygotes diminishes in intensity among individuals with a prolonged or heavy drinking history.^19^ Yokoyama *et al*. first designed a simple two‐question screening tool that asks both current and past alcohol flushing for identifying inactive ALDH2, having reported high sensitivity and specificity in Japanese men (90.1% and 88.0%, respectively).^20^ However, they also showed declined sensitivity of the tool in detecting inactive ALDH2 among moderate‐to‐heavy alcohol consumers, with the sensitivity of 95.4%, 74.3% and 70.4% among never‐to‐light, moderate and heavy consumers, respectively.

Individuals with rs671 AA genotype tend to avoid alcohol consumption due to the very unpleasant responses they experience and are naturally protected from alcohol‐induced carcinogenesis. However, for heterozygous carriers with less severe and gradual tolerance to adverse response, increasing social and cultural pressures put them at greatest EC risk from consuming alcohol. Any use of flushing response as a surrogate biomarker for ALDH2 deficiency is sure to result in a degree of misclassification. The misclassification accounts for the weaker or nonsignificant association of EC with facial flushing than with *ALDH2* genotype, as shown in our study and previous studies.^12^ When using flushing response for screening and prevention counseling, the high false‐negative rate should be particularly cautious among regular alcohol consumers, in which heterozygous carriers has risen substantially in the last few decades.^21^

Our study is thus far the largest prospective study that examined the joint effects of ALDH2 deficiency and alcohol consumption on EC risk. The internal validity of the study was enhanced by prospective design, the exclusion of participants who might lead to reverse causality, and careful adjustment for potential confounders. The inclusion of a geographically spread Chinese population living in urban and rural areas made the findings more generalizable to middle‐aged to older Chinese. Large sample size and incident EC cases allowed better presentation of the dose–response relationship between alcohol consumption and EC by ALDH2 activity.

Some limitations also warrant mention. Alcohol consumption was self‐reported once at baseline. Our study lacked information on histological subtype of EC. However, ESCC accounts for >90% of a subset of EC cases recorded in the CKB population. Despite the overall large sample size, few women consumed alcohol weekly in the present population, precluding the analysis in women. Less than one‐fifth of the CKB participants was genotyped, resulting in wide confidence intervals for the effect estimates of interaction between *ALDH2* genotype and alcohol consumption. It was also hard to consider further the potential influences of other genetic and environmental factors on the EC risk associated with ALDH2 and alcohol consumption.

## Conclusions

In the present male Chinese population, excessive alcohol consumption was associated with an elevated EC risk regardless of ALDH2 activity. However, a noticeably extra increased EC risk related to low‐activity ALDH2 was observed among heavy alcohol consumers, but not among light‐to‐moderate consumers. One of the fundamental measures for EC prevention is refraining from excessive alcohol consumption. It also highlights the importance of identifying high‐risk individuals who have low‐activity ALDH2 and is accustomed to drinking alcohol heavily and providing them with intensive health intervention and early screening for EC in routine clinical practice. Despite low sensitivity in identifying ALDH2 deficiency among regular alcohol consumers, the flushing response remains a simple and noninvasive manner in populations with minimal genotyping resources. However, with the development of rapid and low‐cost methods for genotyping, even the direct‐to‐consumer personal genomic testing has become increasingly popular in some populations. More accurate measurement of increased susceptibility to the carcinogenic effect of alcohol could allow better achievement of personalized precision prevention.

## Contributors

JL and LL conceived and designed the paper. LL, ZC and JC, as the members of CKB steering committee, designed and supervised the conduct of the whole study, obtained funding, and together with YG, ZB, LY, YC, YC, XZ and YL coordinated the data acquisition (for baseline, resurveys and long‐term follow‐up) and standardization. IYM and RGW led the genetic program including DNA extraction and genotyping. CY and JL analyzed the data and drafted the manuscript. JL and LL contributed to the interpretation of the results and critical revision of the manuscript for important intellectual content. All authors contributed to and approved the final manuscript. JL and LL are the study guarantors.

## Supporting information

Supporting Information 1
