# Supplementary material for: Association of low‐activity ALDH2 and alcohol consumption with risk of esophageal cancer in Chinese adults: A population‐based cohort study
Source: Int J Cancer. 2018 Jun 11;143(7):1652–61. doi: 10.1002/ijc.31566 (PMC6175107; doi:10.1002/ijc.31566)
Supplement: Supplementary file 1 — Supporting Information 1 [file IJC-143-1652-s001.docx]

**Supplementary Appendix**

**Table of Contents**

[**Members of the China Kadoorie Biobank collaborative group** 2](#_Toc507179579)

[**Table S1.** Baseline characteristics of eligible male participants 4](#_Toc507179580)

[**Table S2.** *ALDH2* genotype and self-reported flushing response among male weekly alcohol consumers 7](#_Toc507179581)

[**Table S3.** HRs (95% CIs) for joint effects of alcohol consumption with self-reported flushing response on esophageal cancer risk among 162,609 male participants 8](#_Toc507179582)

[**Table S4.** HRs (95% CIs) for joint effects of alcohol consumption with *ALDH2* genotype on esophageal cancer risk among 27,791 male participants 10](#_Toc507179583)

**Members of the China Kadoorie Biobank collaborative group**

**International Steering Committee:** Junshi Chen, Zhengming Chen (PI), Robert Clarke, Rory Collins, Yu Guo, Liming Li (PI), Jun Lv, Richard Peto, Robin Walters.

**International Co-ordinating Centre, Oxford:** Daniel Avery, Ruth Boxall, Derrick Bennett, Yumei Chang, Yiping Chen, Zhengming Chen, Robert Clarke, Huaidong Du, Simon Gilbert, Alex Hacker, Mike Hill, Michael Holmes, Andri Iona, Christiana Kartsonaki, Rene Kerosi, Ling Kong, Om Kurmi, Garry Lancaster, Sarah Lewington, Kuang Lin, John McDonnell, Iona Millwood, Qunhua Nie, Jayakrishnan Radhakrishnan, Paul Ryder, Sam Sansome, Dan Schmidt, Paul Sherliker, Rajani Sohoni, Becky Stevens, Iain Turnbull, Robin Walters, Jenny Wang, Lin Wang, Neil Wright, Ling Yang, Xiaoming Yang.

**National Co-ordinating Centre, Beijing:** Zheng Bian, Yu Guo, Xiao Han, Can Hou, Jun Lv, Pei Pei, Chao Liu, Yunlong Tan, Canqing Yu.

**10 Regional Co-ordinating Centres: Qingdao CDC:** Zengchang Pang, Ruqin Gao, Shanpeng Li, Shaojie Wang, Yongmei Liu, Ranran Du, Yajing Zang, Liang Cheng, Xiaocao Tian, Hua Zhang, Yaoming Zhai, Feng Ning, Xiaohui Sun, Feifei Li. **Licang CDC:** Silu Lv, Junzheng Wang, Wei Hou. **Heilongjiang Provincial CDC:** Mingyuan Zeng, Ge Jiang, Xue Zhou. **Nangang CDC:** Liqiu Yang, Hui He, Bo Yu, Yanjie Li, Qinai Xu,Quan Kang, Ziyan Guo. **Hainan Provincial CDC:** Dan Wang, Ximin Hu, Jinyan Chen, Yan Fu, Zhenwang Fu, Xiaohuan Wang. **Meilan CDC:** Min Weng, Zhendong Guo, Shukuan Wu,Yilei Li, Huimei Li, Zhifang Fu. **Jiangsu Provincial CDC:** Ming Wu, Yonglin Zhou, Jinyi Zhou, Ran Tao, Jie Yang, Jian Su. **Suzhou CDC:** Fang liu, Jun Zhang, Yihe Hu, Yan Lu, , Liangcai Ma, Aiyu Tang, Shuo Zhang, Jianrong Jin, Jingchao Liu. **Guangxi Provincial CDC:** Zhenzhu Tang, Naying Chen, Ying Huang. **Liuzhou CDC:** Mingqiang Li, Jinhuai Meng, Rong Pan, Qilian Jiang, Jian Lan,Yun Liu, Liuping Wei, Liyuan Zhou, Ningyu Chen Ping Wang, Fanwen Meng, Yulu Qin,, Sisi Wang. **Sichuan Provincial CDC:** Xianping Wu, Ningmei Zhang, Xiaofang Chen,Weiwei Zhou. **Pengzhou CDC:** Guojin Luo, Jianguo Li, Xiaofang Chen, Xunfu Zhong, Jiaqiu Liu, Qiang Sun. **Gansu Provincial CDC:** Pengfei Ge, Xiaolan Ren, Caixia Dong. **Maiji CDC:** Hui Zhang, Enke Mao, Xiaoping Wang, Tao Wang, Xi zhang. **Henan Provincial CDC:** Ding Zhang, Gang Zhou, Shixian Feng, Liang Chang, Lei Fan. **Huixian CDC:** Yulian Gao, Tianyou He, Huarong Sun, Pan He, Chen Hu, Xukui Zhang, Huifang Wu, Pan He. **Zhejiang Provincial CDC:** Min Yu, Ruying Hu, Hao Wang. Tongxiang CDC: Yijian Qian, Chunmei Wang, Kaixu Xie, Lingli Chen, Yidan Zhang, Dongxia Pan, Qijun Gu. **Hunan Provincial CDC:** Yuelong Huang, Biyun Chen, Li Yin, Huilin Liu, Zhongxi Fu, Qiaohua Xu. **Liuyang CDC:** Xin Xu, Hao Zhang, Huajun Long, Xianzhi Li, Libo Zhang, Zhe Qiu.

**Table S1. Baseline characteristics of eligible male participants**

|  | **For analysis of flushing response and EC (n=162,609)** | | | | | |  | **For analysis of *ALDH2* genotype and EC (n=29,171)** | | |
| --- | --- | --- | --- | --- | --- | --- | --- | --- | --- | --- |
|  | **Less than weekly** | **Less than daily or <30 g/d** | **≥30 g/d, with flushing response reported as** | | | |  |  |  |  |
|  |  |  | **Soon** | **Small** | **Large** | **No** |  | **GG** | **GA** | **AA** |
| No. of participants, n (%)^*^ | 103,229 | 30,624 | 1,682 | 2,543 | 11,764 | 12,767 |  | 18,168 | 9,623 | 1,380 |
|  | (63.5) | (18.8) | (1.0) | (1.6) | (7.2) | (7.9) |  | (62.3) | (33.0) | (4.7) |
| Age, year | 51.3 | 48.9 | 51.6 | 51.7 | 51.1 | 52.2 |  | 50.8 | 51.0 | 51.5 |
| Rural area, % | 61.6 | 45.2 | 60.4 | 68.2 | 65.0 | 49.3 |  | 61.8 | 58.7 | 56.3 |
| Middle school and above, % | 59.8 | 61.7 | 56.6 | 54.9 | 55.5 | 53.8 |  | 58.4 | 58.2 | 55.3 |
| Married, % | 92.8 | 93.7 | 92.8 | 92.4 | 93.0 | 92.8 |  | 92.9 | 93.2 | 92.7 |
| Household income ≥20,000 RMB per year, % | 45.2 | 48.3 | 45.0 | 43.4 | 45.6 | 41.8 |  | 44.0 | 44.3 | 43.1 |
| Daily smoking, % | 63.2 | 78.0 | 85.9 | 86.1 | 86.5 | 87.6 |  | 71.3 | 70.4 | 68.9 |
| Daily consuming hot/burning hot tea, % | 24.8 | 29.9 | 36.3 | 37.6 | 34.7 | 33.9 |  | 27.8 | 29.7 | 30.3 |
| Physical activity, MET-hour/day | 23.2 | 22.8 | 23.7 | 23.4 | 24.4 | 23.8 |  | 24.0 | 23.6 | 22.8 |
| Average weekly intake, day^†^ |  |  |  |  |  |  |  |  |  |  |
| Red meat | 3.8 | 4.2 | 4.4 | 4.3 | 4.3 | 4.2 |  | 3.9 | 3.9 | 3.8 |
| Fresh fruits | 2.3 | 2.2 | 1.9 | 1.8 | 1.8 | 1.6 |  | 2.1 | 2.3 | 2.4 |
| Fresh vegetables | 6.8 | 6.8 | 6.9 | 6.8 | 6.8 | 6.8 |  | 6.8 | 6.8 | 6.8 |
| Preserved vegetables | 2.0 | 2.2 | 2.6 | 2.7 | 2.5 | 2.5 |  | 2.3 | 2.1 | 2.2 |
| Family history of cancer, % | 16.1 | 16.9 | 18.9 | 17.7 | 18.6 | 17.1 |  | 17.3 | 16.6 | 16.8 |
| Body mass index, kg/m^2^ | 23.2 | 23.4 | 23.3 | 23.4 | 23.2 | 23.0 |  | 23.3 | 23.1 | 23.0 |
| Waist-to-hip ratio | 0.897 | 0.905 | 0.907 | 0.911 | 0.910 | 0.906 |  | 0.904 | 0.895 | 0.893 |
| Daily consuming alcohol, % | -- | -- | -- | -- | -- | -- |  | 32.7 | 8.7 | 0.3 |
| Consumption habits of daily alcohol consumers^‡^ |  |  |  |  |  |  |  |  |  |  |
| Alcohol consumed per day, g | -- | 22.9 | 58.1 | 60.1 | 73.3 | 71.2 |  | 62.9 | 47.8 | 37.0 |
| Duration of alcohol consumption, year | -- | 21.0 | 23.5 | 24.2 | 25.2 | 25.7 |  | 25.1 | 20.4 | 16.2 |
| Usually consuming spirits, % | -- | 47.0 | 89.8 | 87.4 | 84.4 | 84.9 |  | 77.8 | 74.5 | 73.3 |

EC indicates esophageal cancer; and MET, metabolic equivalent of task. The results are presented as adjusted means or percentages, with adjustment for age and study area in analysis of flushing response and adjustment for study area in analysis of *ALDH2* genotype, as appropriate.

^*^The numbers in parentheses indicate the proportion of participants in six categories of alcohol consumption and flushing response or three genotype categories.

^†^Average weekly intake of red meat, fresh fruits and vegetables, and preserved vegetables were calculated by assigning participants to the midpoint of their intake category (daily, 4-6 days/week, 1-3 days/week, monthly, or rarely or never).

^‡^Only daily alcohol consumers were used for calculation.

**Table S2. *ALDH2* genotype and self-reported flushing response among male weekly alcohol consumers**

|  |  | **One-question screening (n=10,706)** | |  | **Two-question screening (n=641)** | | |
| --- | --- | --- | --- | --- | --- | --- | --- |
|  |  | **Flushing**^*^ | **Not flushing** |  | **Current flushing**^*^ | **Former flushing**^†^ | **Never flushing** |
| *ALDH2* genotype | GG | 1,050 | 7,979 |  | 49 | 26 | 457 |
|  | GA | 946 | 717 |  | 58 | 6 | 44 |
|  | AA | 7 | 7 |  | 0 | 0 | 1 |
|  | Total | 2,003 | 8,703 |  | 107 | 32 | 502 |
|  |  |  |  |  |  |  |  |
| Sensitivity^‡^ | | 56.8% | |  | 58.7% | | |
| Specificity^¶^ | | 88.4% | |  | 85.9% | | |

^*^Participants who answered “soon” or “small” to question A were considered to be “current flushing”.

^†^Participants who answered “soon” or “small” to question B but not to question A were considered to be “former flushing.”

^‡^The proportion of participants possessing rs671 A allele who were correctly identified as a flusher by the questionnaire.

^¶^The proportion of participants possessing active rs671 GG genotype who were correctly identified as non-flusher by the questionnaire.

**Table S3. HRs (95% CIs) for joint effects of alcohol consumption with self-reported flushing response on esophageal cancer risk among 162,609 male participants**

|  | **Less than weekly** | **Weekly or <30g/d** | **30-59g/d** | **60-89g/d** | **≥90g/d** |
| --- | --- | --- | --- | --- | --- |
| Cases | 589 | 119 |  |  |  |
| Cases/PYs (/1,000) | 0.64 | 0.43 |  |  |  |
| HRs (95% CIs)^*^ | 1.00 | 1.02 (0.83, 1.25) | -- | -- | -- |
| **No** |  |  |  |  |  |
| Cases |  |  | 42 | 48 | 61 |
| Cases/PYs (/1,000) |  |  | 0.86 | 1.23 | 2.41 |
| HRs (95% CIs)^*^ | -- | -- | 1.54 (1.11, 2.13) | 2.30 (1.68, 3.14) | 4.48 (3.35, 6.00) |
| **Large** |  |  |  |  |  |
| Cases |  |  | 36 | 49 | 70 |
| Cases/PYs (/1,000) |  |  | 0.92 | 1.27 | 2.49 |
| HRs (95% CIs)^*^ | -- | -- | 1.50 (1.06, 2.11) | 2.23 (1.64, 3.04) | 4.44 (3.36, 5.86) |
| **Small** |  |  |  |  |  |
| Cases |  |  | 15 | 13 | 13 |
| Cases/PYs (/1,000) |  |  | 1.43 | 1.39 | 4.19 |
| HRs (95% CIs)^*^ | -- | -- | 2.47 (1.46, 4.18) | 2.30 (1.30, 4.07) | 5.70 (3.21, 10.12) |
| **Soon** |  |  |  |  |  |
| Cases |  |  | 11 | 14 | 10 |
| Cases/PYs (/1,000) |  |  | 1.37 | 2.56 | 6.15 |
| HRs (95% CIs)^*^ | -- | -- | 2.37 (1.29, 4.34) | 4.53 (2.62, 7.84) | 11.73 (6.17, 22.31) |

HR indicates hazard ratio; CI, confidence interval; PYs, person-years; and MET, metabolic equivalent of task.

^*^Multivariable model was adjusted for age (year), education (no formal school, primary school, middle school, high school, college, or university or above), marital status (married, widowed, divorced/separated, or never married), household income (RMB/year: <2,500, 2,500-4,999, 5,000-9,999, 10,000-19,999, 20,000-34,999, or ≥35,000), tobacco smoking (nonsmokers, current smokers 1-9, 10-19, 20-29, or ≥30 cigarettes or equivalents per day), tea consumption and temperature preference (consuming tea less than weekly, weekly, or daily; further categorized into preferring warm, hot, or burning hot tea among daily consumers), physical activity (MET-hour/day), intakes of red meat, fresh fruits and vegetables, and preserved vegetables (day/week, calculated by assigning participants to the midpoint of their intake category), body mass index (kg/m^2^), waist-to-hip ratio, and family history of cancer (presence or absence).

**Table S4. HRs (95% CIs) for joint effects of alcohol consumption with *ALDH2* genotype on esophageal cancer risk among 27,791 male participants**

|  | **Less than weekly** | **Weekly or <30g/d** | **30-59g/d** | **60-89g/d** | **≥90g/d** |
| --- | --- | --- | --- | --- | --- |
| Cases | 110 | 22 |  |  |  |
| Cases/PYs (/1,000) | 0.72 | 0.45 |  |  |  |
| HRs (95% CIs)^*^ | 1.00 | 1.14 (0.71, 1.84) | -- | -- | -- |
| **GG** |  |  |  |  |  |
| Cases |  |  | 16 | 18 | 22 |
| Cases/PYs (/1,000) |  |  | 0.97 | 1.18 | 2.21 |
| HRs (95% CIs)^*^ | -- | -- | 1.96 (1.12, 3.42) | 2.38 (1.35, 4.21) | 4.98 (2.81, 8.83) |
| **GA** |  |  |  |  |  |
| Cases |  |  | 4 | 7 | 5 |
| Cases/PYs (/1,000) |  |  | 1.43 | 4.29 | 6.45 |
| HRs (95% CIs)^*^ | -- | -- | 4.53 (1.58, 13.00) | 15.64 (6.58, 37.17) | 22.54 (8.30, 61.21) |

HR indicates hazard ratio; CI, confidence interval; PYs, person-years; and MET, metabolic equivalent of task.

^*^Multivariable model was adjusted for age (year), education (no formal school, primary school, middle school, high school, college, or university or above), marital status (married, widowed, divorced/separated, or never married), household income (RMB/year: <2,500, 2,500-4,999, 5,000-9,999, 10,000-19,999, 20,000-34,999, or ≥35,000), tobacco smoking (nonsmokers, current smokers 1-9, 10-19, 20-29, or ≥30 cigarettes or equivalents per day), tea consumption and temperature preference (consuming tea less than weekly, weekly, or daily; further categorized into preferring warm, hot, or burning hot tea among daily consumers), physical activity (MET-hour/day), intakes of red meat, fresh fruits and vegetables, and preserved vegetables (day/week, calculated by assigning participants to the midpoint of their intake category), body mass index (kg/m^2^), waist-to-hip ratio, and family history of cancer (presence or absence).
